# Diagnostic Performance of Linked Color Imaging Compared to White Light Imaging During Secondary Endoscopic Evaluation in Patients with Gastric Neoplasia Referred for Endoscopic Resection: A Randomized Comparative Study

**DOI:** 10.5152/tjg.2025.24821

**Published:** 2025-08-11

**Authors:** Jae Gon Lee, In Kyung Yoo, Sang Pyo Lee

**Affiliations:** 1 Department of Internal Medicine, Hanyang University Guri Hospital, Hanyang University College of Medicine, Guri, Korea; 2 Department of Internal Medicine, CHA Bundang Medical Center, CHA University, Seongnam, Korea; 3 Department of Internal Medicine, Hanyang University College of Medicine, Seoul, Korea

**Keywords:** Early gastric cancer, gastroscopy, linked color imaging, stomach neoplasms

## Abstract

**Background/Aims:**

Linked color imaging (LCI) has been shown to improve the visibility of gastric lesions and may enhance the detection of gastric superficial neoplasia (GSN). The aim was to compare the detection performance of LCI versus white light imaging (WLI) in patients referred for endoscopic resection of GSNs.

**Materials and Methods:**

In this randomized prospective comparative study, patients who were referred for endoscopic resection of gastric adenoma or early gastric cancer (ECG) were assigned to either the LCI or WLI group. Initial endoscopic evaluation was performed using the assigned mode (LCI or WLI), followed by a second examination using the alternate mode. The primary outcome was detection sensitivity for GSNs.

**Results:**

Ninety-five patients with 104 lesions were analyzed: 48 in the LCI group and 47 in the WLI group. Detection sensitivity at first observation was 94.23% in the LCI group and 86.54% in the WLI group (*P* = .122). The mean tumor detection time was significantly shorter in the LCI group (54.2 ± 38.4 seconds) than in the WLI group (74.0 ± 55.5 seconds; *P* = .049). Tumors with type IIb morphology were significantly more likely to be missed than tumors with other morphologies (*P* = .014).

**Conclusion:**

Linked color imaging may improve lesion detection performance and efficiency during the endoscopic evaluation of GSNs.

**Main Points**

- Linked color imaging (LCI) demonstrated a tendency to detect gastric superficial neoplasia (GSN) more rapidly and sensitively compared to white light imaging (WLI).
- Although the difference did not reach statistical significance, the detection sensitivity of GSN at the first observation was higher in the LCI group (94.23%) than in the WLI group (86.54%) (*P* = .122).
- The mean tumor detection time was significantly shorter in the LCI group than in the WLI group (54.2 seconds vs. 74.0 seconds, *P* = .049), with particularly improved efficiency observed for lesions located in the gastric body and flat-type (type IIb) morphology.

## Introduction

Gastric cancer is one of the most common cancers worldwide.^1^ Screening endoscopy enables the detection of gastric cancer at an early stage and is therefore crucial in reducing gastric cancer–related deaths.^2^ However, despite the widespread use of screening endoscopy, a significant number of early gastric cancers (EGCs) can be missed.^3^

To increase the diagnosis rates of gastric superficial neoplasms (GSNs), including EGCs and adenomas, various image-enhanced endoscopy (IEE) technologies have been devised.^4^^,^^5^ Linked color imaging (LCI), developed by the Fujifilm Co. (Tokyo) in 2013, increases mucosal color differences by expanding or reducing color information, and studies have shown that LCI is useful for the early detection of EGCs.^6^^-^^8^ In addition, in recent Japanese studies, LCI improved the visibility of gastric cancer significantly after *H*.* pylori* eradication treatment compared to conventional white light imaging (WLI)^9^^,^^10^ and has been shown to increase tumor detection rates in the upper gastrointestinal tract significantly compared to WLI.^11^^,^^12^

When a gastric tumor is detected by primary screening endoscopy and referred for further evaluation and treatment, it is not uncommon for the lesion to be poorly visualized or ambiguous on secondary endoscopic assessment or endoscopic resection. This is especially likely if the lesion is small, flat, and indistinct, its location and morphology are poorly described, or if endoscopic images are of poor quality. Therefore, the aim was to evaluate the detection performance of LCI on secondary endoscopic evaluation in patients referred for the removal of GSNs.

## Materials and Methods

### Patients

This prospective, multi-center, randomized controlled, single-blind, tandem trial was performed from February 2022 to December 2023 at Hallym University Dongtan Sacred Heart Hospital and CHA Bundang Medical Center in Korea. Patients diagnosed with EGC or gastric adenoma and referred for endoscopic evaluation and resection were enrolled in the study. The exclusion criteria were as follows: age <20 years or >90 years, history of gastrectomy, estimated tumor size ≥20 mm at index endoscopy, suspected or diagnosed advanced gastric cancer from the time of referral, and refusal to provide written informed consent. Further, based on the final pathological results of the resected specimen, patients with no adenoma or carcinoma identified or a tumor size ≥20 mm were excluded from the analysis. Patient characteristics, including age, sex, comorbidities, and *H*.* pylori* infection status, were investigated.

This study was conducted with the approval of the Institutional Review Board of Hallym University Dongtan Sacred Heart Hospital and in accordance with the ethical guidelines of the Helsinki Declaration (HDT2022-01-001-002; February 28, 2022). After obtaining IRB approval, this study was registered with the Clinical Research Information Service (CRIS) with ID: KCT0007028.

### Study Groups and Randomization

A researcher who did not otherwise participate in the study used an Excel (Microsoft 365 version 2308; Microsoft Corporation, Redmond, WA, USA) generated random number table to assign patients to the 2 study groups. Patients with an odd number were assigned to the experimental group (the LCI group) and those with an even number to the control group (the WLI group). Group allocation was concealed from the endoscopists until immediately before the endoscopic examination. Endoscopic evaluations for this study were performed by endoscopists who only knew that a certain lesion was present but did not know the characteristics of the lesion or patient information. After the endoscopic evaluation was completed, the endoscopist who referred the patient and had knowledge of the tumor resected all of the referred tumors. Reasons for examinations and details, such as lesion location, were withheld until the endoscopic evaluations had been completed.

Endoscopy was performed by 4 endoscopists with more than 5 years of relevant experience using a Fujifilm upper endoscope (upper endoscope 700 series, Fujifilm Co., Tokyo) at the 2 institutions participating in the study. For patients in either the LCI or WLI groups, the entire stomach was first observed in LCI or WLI mode, respectively. The stomach was inspected in the order of antrum, angle, body, and cardia. Lesions suspected of gastric neoplasia were biopsied (H&E with Giemsa staining), and times taken to detect suspicious lesions were recorded. *H*.* pylori* infection status was initially assessed based on medical history, and the rapid urease test (CLO [Campylobacter-Like Organism] test) was performed for all patients with uncertain infection status. After LCI or WLI evaluations, gastric mucosa was re-examined in the other mode (WLI or LCI, respectively). Gastric observation times for LCI and WLI ranged from 1 to 3 minutes (a minimum of 1 minute to a maximum of 3 minutes) for the first and second examinations, and any lesions newly discovered during the second examinations (WLI or LCI) were biopsied. All lesions originally referred for endoscopic resection were removed using endoscopic submucosal dissection (ESD) method.

### Definitions and Outcome Measures

The size, location, and morphology of the tumors, tumor detection time, endoscopically estimated histologic findings, and final histologic results were evaluated and recorded. Tumor detection time was defined as the time from the start of gastric observation until lesion detection. The tumor size was defined as the length of the longest axis of a specimen as measured by a pathologist after endoscopic resection.

The tumor locations used were pylorus, antrum, angle, body, fundus, cardia along the long axis of the stomach, and anterior wall, posterior wall, greater curvature (GC), and lesser curvature (LC) along the transverse axis. Tumor morphologies were classified as type I (protruded), type IIa (superficial elevated), type IIb (flat), type IIc (superficial depressed), and type III (excavated), as per the Japanese classification of gastric carcinoma.^13^ Tumors with 2 or more components were described based on the proportions of occupied surface area.

Gastric superficial neoplasia (GSN) included EGCs and precancerous lesions.^14^ Therefore, adenoma and adenocarcinoma were considered GSNs, but hyperplastic and inflammatory polyps were not. Gastric lymphoma was not considered GSN in this study.

If a lesion was found during the first or second observations, histological results were classified into low-grade dysplasia, high-grade dysplasia (HGD), mucosal cancer, or submucosal cancer by endoscopist’s discretion using endoscopic macroscopic findings. These results are referred to as “endoscopically estimated histological results.” Final histologic results were derived by reviewing pathologic reports and were classified as adenoma with low- or high-grade dysplasia, mucosal cancer, or submucosal cancer. Rates of diagnostic discrepancy between endoscopic estimation and final histologic results were calculated in each imaging group.

The primary outcome was the detection sensitivity of GSNs in each imaging group. Detection sensitivity was defined as the proportion of the number of detected lesions to the total number of lesions. Secondary outcomes included the tumor characteristics in each group, and the characteristics of the lesions detected or undetected on the first observation.

### Calculation of the Number of Objects

A previous study reported that the detection rates of EGC by WLI and LCI were 35.1% and 69.3%, respectively (missing rate, 64.9% and 30.7%, respectively).^11^ At a 5% level of significance and 90% power, the estimated sample size was 42.714. Thus, assuming a 10% dropout rate, 50patients per group were enrolled .

### Statistical Analysis

The Student’s *t*-test or the Mann–Whitney *U* test and the chi-squared test or Fisher’s exact test were used to determine the significance of intergroup differences between continuous and categorical variables, respectively. The analysis was performed using SPSS, version 19.0 for Windows (IBM SPSS Corp.; Armonk, NY, USA). Results are presented as means ± SD or frequency (%), as appropriate, and statistical significance was accepted for *P* values <.05.

## Results

### Patients and Baseline Characteristics

**Figure 1.**
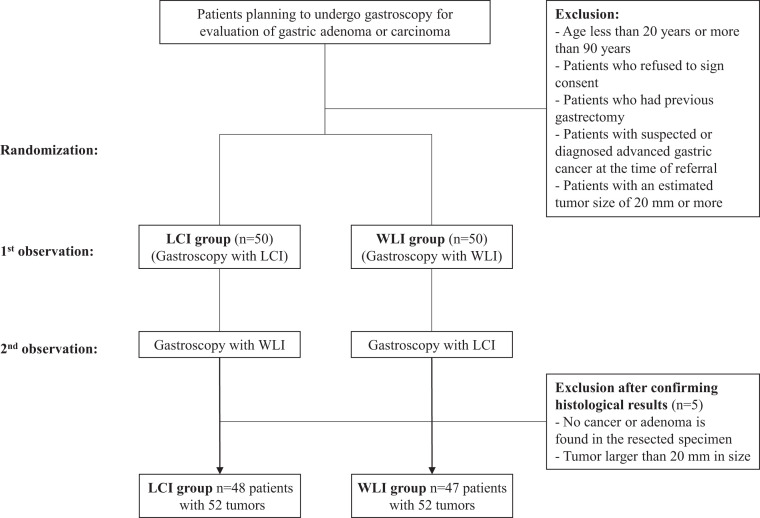
Schematic of the study protocol. LCI, linked color imaging; WLI, white light imaging.

Initially, 100 patients were enrolled (50 per group), but 5 patients were excluded because of a tumor size ≥ 20 mm or the absence of GSN in ESD specimens. Subsequently, 95 patients (48 in the LCI group and 47 in the WLI group) were included (Figure 1). Of the 95 study subjects, 30 (31.6%) were women, and mean ages in the LCI and WLI groups were 63.81 ± 11.22 years (range, 34-86 years) and 64.30 ± 10.73 years (range, 39-87 years), respectively. No significant difference was found between the 2 groups in terms of age, sex, underlying diseases, medication history, or *H*.* pylori* infection status (Table 1). Endoscopy was performed by 4 expert endoscopists. Endoscopist assignment, number of biopsies performed, and the number of GSNs found were not significantly different between the 2 groups (Table 2).

### Detection Sensitivity and Characteristics of Gastric Superficial Neoplasia

A total of 97 gastric neoplastic lesions from 95 patients were initially referred. During the study examinations, 7 additional lesions were newly identified, resulting in a total of 104 lesions. Table 3 summarizes the detection sensitivity and characteristics of GSNs. At the first observation, 3 of 52 tumors in the LCI group and 7 of 52 tumors in the WLI group were not detected. Thus, the detection sensitivity for GSNs at first observation was 94.23% (49 of 52 tumors) in the LCI group and 86.54% (45 of 52 tumors) in the WLI group (*P* = .122). Tumor sizes, locations, morphologies, and final histopathologic types were not significantly different between the groups. Among the 7 tumors missed at the first observation in the WLI group, 3 were subsequently detected during the second observation using LCI. In contrast, none of the 3 missed tumors in the LCI group were detected during the second observation with WLI. The characteristics of the 7 newly detected lesions, which were not among those originally referred, are presented in Supplementary Table 1.

Subgroup analysis of tumor detection rate based on tumor location demonstrated no statistically significant differences in detection rates between LCI and WLI across both longitudinal (body, antrum, angle, cardia) and transverse (LC, GC, posterior wall, anterior wall) axes. Although the differences were not statistically significant, detection rates were numerically higher with LCI across all tumor locations along the longitudinal axis. Along the transverse axis, LCI also demonstrated higher detection rates at all sites except for the LC (Supplementary Table 2).

The mean tumor detection time in the LCI group was 54.20 (± 38.43) seconds, which was significantly shorter than the 74.02 (± 55.47) seconds in the WLI group ( *P* = .049). There were also significantly fewer cases of tumor detection times longer than 1 minute in the LCI group (18 in the LCI group and 27 in the WLI group, *P* = .038).

Subgroup analysis of tumor detection time based on tumor characteristics revealed that the LCI group demonstrated a shorter detection time for tumors located in the body (64.3 ± 36.2 seconds vs. 122.4 ± 54.5 seconds, *P* = .003). For tumor morphology, the detection time for flat lesions (IIb) was shorter in the LCI group than in the WLI group (65.1 ± 45.0 seconds vs. 96.7 ± 64.7 seconds, *P* = .148) (Supplementary Table 3).

### Undetected and Detected Tumors at First Observation

**Figure 2.**
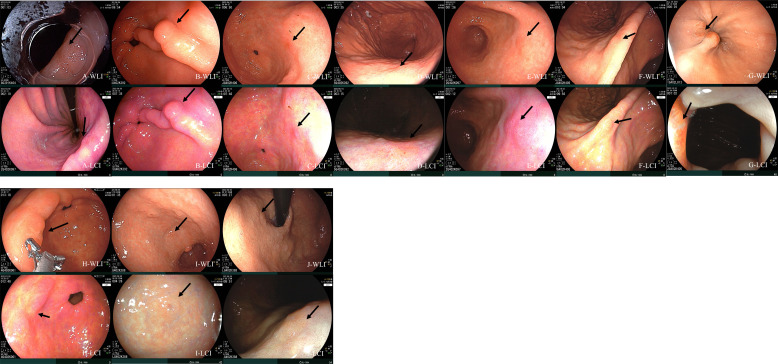
Gastric superficial neoplasms that were not detected at first observation. The 7 cases in the top row of the figure above (A to G) are cases in which no lesions were found during the first or second observations, and the 3 cases in the bottom row (H to J) are cases in which no lesions were found during the first observation, but lesions were found during the second observation. Cases A, B, and C were in the LCI group, and the others were in the WLI group. In all cases, tumor sizes and histological findings were confirmed by histopathology of ESD specimens. A. This IIb tumor, located at the LC side of the lower body, was histologically proven to be mucosal adenocarcinoma and had a confirmed tumor size of 9 mm after ESD. B. This IIa tumor was located on the posterior wall side of the antrum and had a histological finding of adenoma with low-grade dysplasia (LGD) and a tumor size of 3 mm. C. This IIb tumor, located at the posterior wall side of the antrum, was an adenoma with LGD and a tumor size of 2 mm. D. This IIb tumor, located at the posterior wall side of the high body, was an adenoma with HGD and a tumor size of 6 mm. E. This IIb tumor, located at the posterior wall side of the antrum, was an adenoma with HGD and a tumor size of 6 mm. F. This IIb tumor, located at the anterior wall side of the angle, was an adenoma with LGD and a tumor size of 3 mm. G. This IIb tumor, located on the GC side of the cardia, was an adenoma with LGD and a tumor size of 3 mm. H. This IIb+IIc tumor, located at the anterior wall side of the antrum, was an adenoma with LGD and a tumor size of 13 mm. I. This IIb+IIa tumor, located at the LC side of the antrum, was an adenoma with LGD and a tumor size of 7 mm. J. This IIb tumor, located at the posterior wall side of the cardia, was an adenoma with HGD and a tumor size of 8 mm. ESD, endoscopic submucosal dissection; LC, lesser curvature; GC, greater curvature; LGD, low-grade dysplasia; HGD, high-grade dysplasia.

The morphologies of tumors detected and not detected at first observation differed significantly (Table 4, *P* = .014). Undetected tumors were significantly more likely to be type IIb; that is, type IIb lesions accounted for 90% of undetected tumors but only 29.8% of detected tumors. No significant differences were observed between tumors detected or not at first observation in terms of tumor size, tumor location, final histopathologic type, *H*.* pylori* infection status, image mode, age, sex, and endoscopist. However, although not statistically significant, undetected tumors were smaller than detected tumors (mean tumor size, 6.00 mm vs. 8.16 mm, respectively), more likely to be located in the cardia (gastric cardia location, 20.0% vs. 1.1%, respectively), and more likely to be associated with *H*.* pylori* eradication (past *H*.* pylori* infection, 60% vs. 36.2%, respectively). Additional data concerning the 10 tumors not detected at first observation are shown in Figure 2.

### Consistency of Endoscopically Estimated and Final Histologic Results

Based on the revised Vienna classification, final pathologic results were adenoma with low-grade dysplasia, adenoma with HGD, and adenocarcinoma in 23, 15, and 14 cases, respectively, in the LCI group, and in 27, 17, and 8 cases in the WLI group (Table 5). Diagnostic discrepancy rates were similar at 38.8% in the LCI group and 40.0% in the WLI group. Diagnosis upgrade and downgrade rates were 30.6% and 8.2% in the LCI group and 33.3% and 6.7% in the WLI group, respectively.

## Discussion

Our results show that the detection sensitivity for GSNs was higher in the LCI group than in the WLI group (94.23% vs. 86.54%, respectively), although the difference was not statistically significant. The fact that 13.46% of lesions were still missed during standard WLI endoscopy, even though the endoscopist was aware that the lesion was present somewhere in the stomach, highlights a limitation of standard endoscopic examination. Endoscopists should be aware of this limitation and ensure meticulous inspection to minimize the risk of missing lesions.

One of the enhanced imaging modalities of the Fujifilm endoscopy system, blue laser imaging (BLI), enhances the 410 nm blue-violet wavelength to improve the visualization of blood vessels and surface patterns. The BLI-bright mode further adjusts the white light component to produce brighter images. LCI is generated from the BLI-bright mode through digital image processing, which amplifies the intensity of reds and whites to enhance color contrast.^15^ This process enables LCI to produce bright and high-color contrast images, enhancing the visualization of subtle mucosal color changes. By increasing the color contrast between neoplastic and non-neoplastic areas, LCI improves the visibility of upper gastrointestinal tract neoplastic lesions. ^7^^, ^^10^ In the re sults, 3 of the 7 tumors that were missed during the first observation with WLI were subsequently detected during the second observation with LCI. In contrast, all 3 tumors missed during the first observation with LCI were also missed during the second observation with WLI, suggesting that LCI provides better visibility of gastric lesions.

A recent systematic review and meta-analysis by Duan et al,^16^ including 11 studies with 7836 patients, reported that the detection rates of EGC were significantly higher with LCI (85%) compared to WLI (56.7%). Additionally, a study focusing on the detection of EGCs in patients after *H*.* pylori* eradication showed a markedly lower miss rate of EGCs with LCI (30.7%) compared to WLI (64.9%).^11^ In this study, the detection rate of gastric neoplasia was 94.23% with LCI and 86.54% with WLI, which is higher than the rates reported in a previous meta-analysis. This discrepancy may be attributed to the study design, as endoscopists were already aware of the presence of gastric neoplasia and actively searched for lesions. These findings suggest that while LCI can improve detection rates of gastric neoplasia, the context of endoscopic evaluation may influence the outcomes.

In this study, flat lesions (type IIb) demonstrated a higher likelihood of being missed compared to other morphologies. Flat lesions are inherently difficult to detect due to the absence of mucosal protrusion or depression, making it challenging to determine their size and pathological features during endoscopic evaluation. Since LCI enhances mucosal color contrast, it is expected to improve the detection of flat lesions that lack distinct protrusion or depression. In the subgroup analysis of detection time, flat lesions in the LCI group exhibited numerically shorter detection times compared to the WLI group, although the difference did not reach statistical significance. This suggests that LCI may have the potential to enhance the detection of flat lesions, warranting further studies with larger sample sizes to confirm this observation.

The observation time was set for both the first and second examinations at 1-3 minutes, considering that the cumulative observation time could extend up to 6 minutes due to the 2 separate evaluations. This approach also aimed to assess how efficiently lesions could be detected within a limited timeframe. Previous studies on interval gastric cancer have reported an increased risk of missed cancers with observation times of less than 3 minutes.^17^ Therefore, it is possible that longer observation times may further enhance detection rates. Future studies should evaluate the differences in detection rates based on varying observation durations to better understand the impact of observation time on lesion detection.

Several studies have shown that the posterior wall is a potential blind spot for endoscopic observations^18^ and that gastric adenomas and cancers are difficult to detect in the stomach after *H*.* pylori *eradication.^19^^,^^20^ These results also demonstrated that undetected tumors were more often located in the gastric posterior wall and more frequently observed in patients who had undergone *H*.* pylori* eradication therapy, although the differences were not significant. Large-scale follow-up studies may reveal statistical differences*. H*.* pylori* eradication therapy is now widely implemented in real-world clinical practice, and evidence supports a reduced risk of gastric cancer after eradication.^21^^,^^22^ However, gastric cancer still occurs after *H*.* pylori *eradication, and post-eradication gastric cancer is difficult to detect and diagnose because it is likely to be covered by non-cancerous epithelium.^23^ Therefore, surveillance endoscopy after *H*.* pylori *eradication should be performed more rigorously, and the use of IEE should be actively considered to improve lesion detection.

In the results, LCI demonstrated a shorter tumor detection time compared to WLI, with a particularly notable difference observed for tumors located in the gastric body. Sufficient endoscopic observation time and an appropriate screening interval time can reduce the incidence of interval gastric cancer.^18^ However, as screening endoscopy is often performed within a limited timeframe, the shorter detection time with LCI suggests its potential to facilitate tumor detection during screening procedures. Furthermore, since the gastric body is typically observed from a distant view, the enhanced detection performance of LCI in this region may be particularly beneficial. Endoscopists should familiarize themselves with the macroscopic appearance of gastric tumors under LCI inspection.^18^^,^^24^^,^^25^

Discrepancy rates between endoscopic and pathologic diagnosis in the LCI and WLI groups were similar at 38.8% and 40.0%, respectively. Therefore, it was believed that LCI helps detect lesions but is not superior to WLI for the presumptive histological diagnosis of lesions. Rather, BLI or narrow-band imaging, with or without magnification, might be more useful for characterizing lesions and presumptive histologic diagnoses. Since there was no magnifying endoscopy in this study, additional studies are needed to determine whether the use of LCI with magnification might change the results.

This study has some limitations that warrant consideration. First, although the endoscopic examination time was limited, the lesion detection rates observed in the results may differ from true detection rates in real-world endoscopy practice, as the endoscopists were aware of the presence of tumors. Therefore, the generalizability of the findings to routine clinical practice may be limited. Further, the sample size calculation was based on previous research where endoscopists were unaware of the presence of tumors, which may have led to the study being underpowered. Although some differences did not reach statistical significance, the observed trends suggest potential clinical relevance, warranting further large-scale studies. Second, patients were not evenly distributed across the 4 endoscopists and the sample size was too small to adjust biases. Additionally, this study included a limited number of endoscopists, which precluded analysis of potential differences in the diagnostic performance of LCI according to the endoscopists’ experience. Third, although CLO testing was performed on all patients with an uncertain *H*.* pylori* infection status, the omission of additional diagnostic methods for *H*.* pylori* infection, such as histology or serology, is a limitation of this study. Relying solely on a single CLO test may have led to inaccuracies in determining *H*.* pylori* infection status, potentially influencing the study results. This limitation should be carefully considered when interpreting the findings.

In conclusion, although the difference in detection sensitivity between LCI and WLI did not reach statistical significance, LCI demonstrated a numerically higher detection rate and significantly shorter detection time. These findings suggest that LCI may have potential utility in improving the efficiency of lesion detection during upper gastrointestinal endoscopy, particularly in identifying subtle or flat lesions.

## Supplementary Materials

Supplementary Material

## Figures and Tables

**Table 1.** Baseline Characteristics of the Patients

Variables	LCI Group (n = 48)	WLI Group (n = 47)	*P*
Age*, years, mean (±SD)	63.81 (±11.22)	64.30 (±10.73)	.830
Age range, years	34-86	39-87	
≥60, n (%)	32 (66.7)	31 (66.0)	1.000
Female sex, n (%)	17 (35.4)	13 (27.7)	.509
Comorbidity, n (%)	32 (66.7)	30 (63.8)	.831
Diabetes	14 (29.2)	12 (25.5)	.819
Hypertension	25 (52.1)	24 (51.1)	1.000
Dyslipidemia	13 (27.1)	11 (23.4)	.814
Chronic obstructive pulmonary disease	1 (2.1)	2 (4.3)	.617
Congestive heart disease	7 (14.6)	5 (10.6)	.759
Chronic kidney disease	1 (2.1)	2 (4.3)	.617
Stroke	3 (6.3)	2 (4.3)	1.000
Antithrombotic agent, n (%)			
Antiplatelet agent	10 (20.8)	5 (10.6)	.261
Aspirin	3	2	
Clopidogrel	6	1	
Aspirin plus clopidogrel	1	2	
Anticoagulant	0	0	
*H*.* pylori* infection, n (%)			.358
*H*.* pylori*–positive	19 (39.6)	20 (43.5)	
Eradicated	16 (33.3)	19 (41.3)	
Uninfected or remote past infection	13 (27.1)	7 (15.2)	
Patients with 2 referred lesions, n (%)	0	2 (4.3)	.242

Categorical data are presented as numbers (%) and analyzed by the chi-square test and Fisher’s exact test.

*H*.* pylori*,* Helicobacter pylori*; LCI, linked color imaging; WLI, white light imaging.

*Continuous variables arre summarized as mean ± SD and analyzed by the Student *t*-test.

**Table 2.** Endoscopist Distribution and Lesion Detection Outcomes in the Linked Color Imaging and White Light Imaging Groups

Variables	LCI Group (n = 48)	WLI Group (n = 47)	*P*
Endoscopist, n (%)			.681
A	18 (37.5)	14 (29.8)	
B	23 (47.9)	27 (57.4)	
C	6 (12.5)	4 (8.5)	
D	1 (2.1)	2 (4.3)	
Number of lesions for which biopsies were performed, n (%)			.388
0	0	2 (4.3)	
1	34 (70.8)	36 (76.6)	
2	12 (25.0)	7 (14.9)	
3	2 (4.2)	2 (4.3)	
Number of GSNs found during the first and second observations, n (%)			.877
0	3 (6.3)	4 (8.5)	
1	41 (85.4)	39 (83.0)	
2	4 (8.3)	3 (6.4)	
3	0	1 (2.1)	

All the data are presented as numbers (%) and analyzed by the chi-square test or Fisher’s exact test. Gastric superficial neoplasia includes early gastric cancers and precancerous lesions. Therefore, adenoma and adenocarcinoma are applicable, but hyperplastic polyp and inflammatory polyp are not.

GSN, gastric superficial neoplasia; LCI, linked color imaging; WLI, white light imaging.

**Table 3.** Detection Sensitivity and Characteristics of Gastric Superficial Neoplasias

Variables	LCI Group	WLI Group	*P*
Total number of GSNs	52	52	
Number of neoplasms referred from other hospitals	48	49	
Number of neoplasms additionally found during the first and second observations	4	3	
Number of GSNs missed during first observation	3	7	.122
Number of GSNs missed during first and second observations	3	4	1.000
Detection sensitivity of GSNs, %	**94.23**	**86.54**	.122
Tumor detection time*^†^, seconds, mean (±SD)	54.20 (±38.43)	74.02 (±55.47)	**.049**
≥60 sec	18 (36.7)	27 (60.0)	**.038**
Tumor size*, mm, mean (±SD)	8.75 (±5.28)	7.15 (±4.34)	.095
≥10 mm	20 (38.5)	13 (25.0)	.206
≤5 mm	15 (28.8)	19 (36.5)	.531
Tumor location			
Longitudinal axis, n (%)			.430
Antrum	26 (50.0)	33 (63.5)	
Body	18 (34.6)	11 (21.2)	
Cardia	1 (1.9)	2 (3.8)	
Angle	7 (13.5)	6 (11.5)	
Transverse axis, n (%)			.855
Lesser curvature	17 (32.7)	21 (40.4)	
Greater curvature	11 (21.2)	9 (17.3)	
Anterior wall	12 (23.1)	12 (23.1)	
Posterior wall	12 (23.1)	10 (19.2)	
Tumor morphology, n (%)			.289
Type 0-IIa	34 (65.4)	27 (51.9)	
IIa/IIa+IIb/IIa+IIc	16/7/11	18/3/6	
Type 0-IIb	15 (28.8)	22 (42.3)	
IIb/IIb+IIa/IIb+IIc	8/1/6	15/2/5	
Type 0-IIc	3 (5.8)	3 (5.8)	
IIc/IIc+IIb	2/1	0/3	
Final histopathology, n (%)			.409
Adenoma with LGD	23 (44.2)	27 (51.9)	
Adenoma with HGD	15 (28.8)	17 (32.7)	
Adenocarcinoma, mucosal cancer	12 (23.1)	8 (15.4)	
Adenocarcinoma, SM cancer	2 (3.8)	0	

GSN, gastric superficial neoplasia; HGD, high-grade dysplasia; LCI, linked color imaging; LGD, low-grade dysplasia; SM cancer, submucosal cancer; WLI, white light imaging.

*Continuous variables are summarized as mean ± SD and analyzed with Student’s *t*-test or the Mann‒Whitney *U* test. All other data are presented as numbers (%) and analyzed by the chi-square test or Fisher’s exact test.

^†^All tumor detection times for tumors not found in the first observation were treated as missing values.

Statistically significant values are marked in bold.

**Table 4.** Comparison Between Undetected and Detected Tumors at First Observation

Variables	Undetected Tumor (n = 10)	Detected Tumor (n = 94)	*P*
Tumor size*, mm, mean (±SD)	6.00 (±3.43)	8.16 (±4.97)	.095
≥10 mm	1 (10.0)	32 (34.0)	.164
≤5 mm	4 (40.0)	30 (31.9)	.725
Tumor location			
Longitudinal axis, n (%)			.054
Antrum	5 (50.0)	54 (57.4)	
Body	2 (20.0)	27 (28.7)	
Cardia	2 (20.0)	1 (1.1)	
Angle	1 (10.0)	12 (12.8)	
Transverse axis, n (%)			.172
Lesser curvature	2 (20.0)	36 (38.3)	
Greater curvature	1 (10.0)	19 (20.2)	
Anterior wall	2 (20.0)	22 (23.4)	
Posterior wall	5 (50.0)	17 (18.1)	
Tumor morphology, n (%)			**.014**
Type 0-IIa	1 (10.0)	60 (63.8)	
IIa/IIa+IIb/IIa+IIc	1/0/0	33/10/17	
Type 0-IIb	9 (90.0)	28 (29.8)	
IIb/IIb+IIa/IIb+IIc	7/1/1	16/2/10	
Type 0-IIc	0	6 (6.4)	
IIc/IIc+IIb	0	2/4	
Final histopathology, n (%)			.924
Adenoma with LGD	6 (60.0)	44 (46.8)	
Adenoma with HGD	3 (30.0)	29 (30.9)	
Adenocarcinoma, mucosal cancer	1 (10.0)	19 (20.2)	
Adenocarcinoma, SM cancer	0	2 (2.1)	
*H*.* pylori* infection, n (%)			.387
*H*.* pylori*–positive	3 (30.0)	40 (42.6)	
Eradicated	6 (60.0)	34 (36.2)	
Uninfected or remote past infection	1 (10.0)	20 (21.3)	
Image mode for first observation, n (%)			.319
LCI	3 (30.0)	49 (52.1)	
WLI	7 (70.0)	45 (47.9)	
Age*, years, mean (±SD)	62.60 (±10.82)	64.60 (±10.79)	.590
Female sex, n (%)	4 (40.0)	30 (31.9)	.725
Endoscopist, n (%)			.589
A	5 (50.0)	30 (31.9)	
B	5 (50.0)	50 (53.2)	
C	0	11 (11.7)	
D	0	3 (3.2)	

HGD, high-grade dysplasia; LCI, linked color imaging; LGD, low-grade dysplasia; SM cancer, submucosal cancer; WLI, white light imaging.

*Continuous variables are summarized as mean ± SD and analyzed with the Mann‒Whitney *U* test. All other data are presented as numbers (%) and analyzed by the chi-square test or Fisher’s exact test.

**Table 5.** Consistency Between Endoscopically Estimated Histologic Result and Final Histologic Result (n = 187)

Variables	GSNs in the LCI Group	GSNs in the WLI Group	*P*
Histologic results estimated by endoscopic findings^†^, n (%)			
Adenoma with low-grade dysplasia	30 (61.2)	32 (71.1)	
Adenoma with high-grade dysplasia	12 (24.5)	10 (22.2)	
Adenocarcinoma	7 (14.3)	3 (6.7)	
Final histologic results, n (%)			
Adenoma with low-grade dysplasia	23 (44.2)	27 (51.9)	
Adenoma with high-grade dysplasia	15 (28.8)	17 (32.7)	
Adenocarcinoma	14 (26.9)	8 (15.4)	
Discrepancy between the 2 results, n (%)			.949
No change in the result	30 (61.2)	27 (60.0)	
Upgraded diagnosis	15 (30.6)	15 (33.3)	
Downgrade diagnosis	4 (8.2)	3 (6.7)	

All data are presented as numbers (%) and analyzed by Fisher’s exact tests.

^†^Estimated histologic results for tumors not found in the first observation were treated as missing values.

## Data Availability

The data that support the findings of this study are available on request from the corresponding author.
