# Supplementary material for: Diagnostic Performance of Linked Color Imaging Compared to White Light Imaging During Secondary Endoscopic Evaluation in Patients with Gastric Neoplasia Referred for Endoscopic Resection: A Randomized Comparative Study
Source: Turk J Gastroenterol. 2025 Aug 11;37(1):44–54. doi: 10.5152/tjg.2025.24821 (PMC12824881; doi:10.5152/tjg.2025.24821)
Supplement: Supplementary Material [file supplementary_material.pdf]

Supplementary Table 1. Characteristics of lesions newly detected during the study that were not among those originally referred for resection

Case no.	Group	Mode of detection	Location	Size (mm)	Morphology	Final pathology
1	LCI	LCI	Body, LC	18	IIb	Adenoma with HGD
2	LCI	LCI	Body, PW	14	IIc+IIb	Mucosal cancer
3	WLI	WLI	Antrum, GC	10	IIa+IIc	Adenoma with LGD
4	WLI	LCI	Antrum, AW	7	IIb+IIc	Adenoma with LGD
5	WLI	WLI	Antrum, GC	6	IIc+IIb	Adenoma with LGD
6	LCI	LCI	Body, GC	10	IIa+IIb	Adenoma with LGD
7	LCI	LCI	Body, PW	3	IIa+IIb	Adenoma with LGD

LCI, linked color imaging; WLI, white light imaging; HGD, high-grade dysplasia; LGD, low-grade dysplasia; LC, lesser curvature; GC, greater curvature; AW, anterior wall; PW, posterior wall.

Supplementary Table 2. Subgroup analysis of tumor detection rate based on tumor location

	Group	Detection rate, % (n)	*P*-value
Longitudinal axis			
Body	LCI group (n=18)	94.4 (17/18)	1.000
	WLI group (n=11)	90.9 (10/11)	
Antrum	LCI group (n=26)	92.3 (24/26)	1.000
	WLI group (n=33)	90.9 (30/33)	
Angle	LCI group (n=7)	100 (7/7)	0.936
	WLI group (n=6)	83.3 (5/6)	
Cardia	LCI group (n=1)	100 (1/1)	0.665
	WLI group (n=2)	0 (0/2)	
Transverse axis			
Lesser curvature	LCI group (n=17)	94.1 (16/17)	1.000
	WLI group (n=21)	95.2 (20/21)	
Greater curvature	LCI group (n=11)	100 (11/11)	0.918
	WLI group (n=9)	88.9 (8/9)	
Anterior wall	LCI group (n=12)	100 (12/12)	0.460
	WLI group (n=12)	83.3 (10/12)	
Posterior wall	LCI group (n=12)	83.3 (10/12)	0.816
	WLI group (n=10)	70.0 (7/10)	

LCI, linked color imaging; WLI, white light imaging.

Supplementary Table 3. Subgroup analysis of tumor detection time according to tumor location and morphology (detected lesions only; n=94)

	Group	Detection time (seconds, mean ± SD)	*P*-value
Tumor location			
Body	LCI group (n=17)	64.3 ± 36.2	0.003
	WLI group (n=10)	122.4 (54.5	
Antrum	LCI group (n=24)	54.9 ± 42.1	0.530
	WLI group (n=30)	63.0 ± 50.5	
Angle	LCI group (n=7)	34.4 ± 19.9	0.524
	WLI group (n=5)	43.4 ± 27.5	
Cardia	LCI group (n=1)	5.0 (N/A)	N/A
	WLI group (n=0)	N/A	
Tumor morphology			
Flat elevated (type IIa)	LCI group (n=33)	49.7 ± 36.4	0.155
	WLI group (n=27)	65.6 ± 49.0	
Flat (type IIb)	LCI group (n=13)	65.1 ± 45.0	0.148
	WLI group (n=15)	96.7 ± 64.1	
Flat depressed (type IIc)	LCI group (n=3)	57.0 ± 30.2	0.447
	WLI group (n=3)	36.7 ± 28.9	

LCI, linked color imaging; WLI, white light imaging; SD, standard deviation; N/A, not applicable.
